# Editorial discourses in the history of Acta Paulista de Enfermagem (1988-2017)

**DOI:** 10.1590/0034-7167-2023-0362

**Published:** 2024-12-16

**Authors:** Ricardo Quintão Vieira, Alexandre Pazetto Balsanelli, Vanessa Ribeiro Neves

**Affiliations:** I Universidade Federal de São Paulo. São Paulo, São Paulo, Brazil

**Keywords:** History of Nursing, Periodical, Address, Publishing, Social Control, Informal., Historia de la Enfermería, Publicación Periódica, Discurso, Edición, Controles Informales de la Sociedad.

## Abstract

**Objectives:**

to analyze the editorial discourses of Acta Paulista de Enfermagem from 1988 to 2017.

**Methods:**

qualitative, historical, oral research, with interviews with the journal’s editors. Statements were categorized and presented in three decades, discussed from Foucault’s archaeological perspective.

**Results:**

seven statements presented three discourses. In the first decade, the discourse of knowledge registration and circulation presented statements of graduate studies and professional recognition. In the second decade, knowledge internationalization was added, with statements of business and editorial panopticism, selection criteria, indexing and digitalization. Finally, the discourse of shifting scientific assessment centrality was added with statements of preprint, open science, exclusive digitalization and mediatization of science.

**Final Considerations:**

the journal needed to adapt to form its official discourse, which made it possible, over the years, to change its initial peripheral position to a central one within scientific communication, supporting its panoptic role.

## RESUMEN

**Objetivos:**

analizar los discursos editoriales de Acta Paulista de Enfermagem de 1988 a 2017.

**Métodos:**

investigación cualitativa, histórica, oral, con entrevistas a los editores de la revista. Los enunciados fueron categorizados y presentados en tres décadas, discutidos desde la perspectiva arqueológica de Foucault.

**Resultados:**

siete testimonios trajeron tres discursos. En la primera década, el discurso del registro y la circulación del conocimiento presentó declaraciones de posgrado y reconocimiento profesional. La segunda década se sumó a la internacionalización del conocimiento, con declaraciones empresariales y panopticismo editorial, criterios de selección, indexación y digitalización. Finalmente, al discurso de desplazamiento de la centralidad de la evaluación científica se sumó afirmaciones sobre *preprint*, ciencia abierta, digitalización excluyente y mediatización de la ciencia.

**Consideraciones Finales:**

la revista necesitó adaptarse para formar su discurso oficial, lo que permitió, con el paso de los años, cambiar su posición periférica inicial a la central dentro de la comunicación científica, corroborando su papel panóptico.

## INTRODUCTION

Scientific journals are the main pillars of dissemination of modern science, due to their power to disseminate knowledge and promote the development of scientific literature in an exponential and rapid manner^(1)^. In the field of nursing, these communication resources are currently the most cited document formats in international literature^(2)^.

Historically, The American Journal of Nursing is considered the first global nursing journal using the current publishing and printing models, having been created in 1900, in the United States of America^(3)^. In turn, the *Revista Annaes de Enfermagem* represented a Brazilian editorial experience since the 1930s, and today is known as the *Revista Brasileira de Enfermagem* (REBEn), being the oldest nursing journal in force in the national context.

Soon after the creation of the *Escola de Enfermeiras do Departamento Nacional de Saúde Pública* in 1923, today called the *Escola de Enfermagem Anna Nery*, in Rio de Janeiro, American nurses instilled in the novice Brazilian professional nurses the idea that a journal would advance the profession^(4)^.

This historic journal maintained an exclusive position as a vehicle for publicizing academic institutions until 1967, when the *Universidade de São Paulo* launched the first issue of the *Revista da Escola de Enfermagem da USP* (REEUSP), followed, years later, by the *Revista Gaúcha de Enfermagem* (RGE), from the *Universidade Federal do Rio Grande do Sul*, in 1976.

In the 1980s, two nursing journals were launched: at the *Universidade Federal da Bahia*, in 1981, called *Revista Baiana de Enfermagem* (RBE), followed, in 1988, by *Acta Paulista de Enfermagem* (APE), by the *Escola Paulista de Medicina* Department of Nursing (EPM-DN), now the *Escola Paulista de Enfermagem* (EPE) of the *Universidade Federal de São Paulo* (UNIFESP), which highlighted the fact that there have been, since then, two nursing journals in the capital of São Paulo.

APE aims to publish research results and improve care practices in different healthcare contexts, forming part of EPE’s Internal Regulations^(5)^. During its growth and maturation as a vehicle for scientific communication in nursing over the decades, APE has developed a current discourse based on quality criteria and editorial goals for the pursuit of excellence in scientific dissemination, such as indexing in global access databases (PubMed Central and MEDLINE), achieving A1 stratification in the Coordination for the Improvement of Higher Education Personnel (In Portuguese, CAPES - *Coordenação de Aperfeiçoamento de Pessoal de Nível Superior*) Qualis, increasing impact factor, “Continuous Publication”, consolidation of RevEnf Portal and adherence to preprint^(6)^.

Discourses presented by APE, and not only by this journal, but by all journal editors who seek and strive to achieve and maintain quality criteria, sociologically represent the maintenance of the norms and rules of science as a whole.

Discourse is any communicative activity that brings meaning between interlocutors in their interactive relationships. To think about discourse, it is necessary to understand that there is a social place where a discourse is constructed by whom it is said, to whom it is said, and its coexistence with other discourses that circulate socially^(7)^. From this perspective, the official history, especially official discourse, brings important elements for understanding APE’s path as a vehicle of scientific information, but presents limitations for theoretical and sociological discussion, a fact that impoverishes the depth of discursive formations that are hidden among its members.

Given this theoretical framework, and accepting the premise that journals, especially scientific editors, participate in scientific communication and are influenced by the forces that support the social and scientific controls of nursing literature, the question was raised as to how editorial discourses were produced by APE from 1988 to 2017.

## OBJECTIVES

To analyze the editorial discourses developed by APE from 1988 to 2017.

## METHODS

### Ethical aspects

The project was submitted for consideration by the UNIFESP Research Ethics Committee, in accordance with the recommendations of the Brazilian National Health Council Resolution 466/12 of the Ministry of Health, with approval for conducting the research in August 2019. For data collection, participants’ consent was requested through the Informed Consent Form, in compliance with the recommendations of Circular Letter 2/2021/CONEP/SECNS/MoH of April 24, 2021 called “Guidelines for procedures in research with any stage in a virtual environment”.

### Study design

This is a historical study that consists of observing and qualitatively describing the social relationship established between man and their environment, the effects of which can be observed at the present time, under the complex range of visions, approaches and perspectives. In this study, oral history was chosen based on recorded and treated oral sources. Moreover, these sources were analyzed in the light of Archaeology of Knowledge, focused on discursive formations (discursive and non-discursive elements).

In order to comply with the reproducibility process, this research can be summarized in the following stages: study object historical survey; justified time frame; research setting selection; participants (with inclusion and exclusion criteria); interview scheduling; audio (or video) recording using a semi-structured questionnaire; conversion of oral speech to written speech (transcription, textualization, transcreation); coding; categorization; presentation of results with discourse analysis (“buried” discursive formations); discussion of results with updated literature according to “unearthed” (exposed) discursive elements.

The checklist proposed by the COnsolidated criteria for REporting Qualitative research (COREQ): a 32-item checklist for interviews and focus groups was used in all its domains: research team and reflexivity; study design; data analysis.

### Methodological procedures

Time frame covered the period between 1988, the year in which the first publication of APE occurred, a milestone in the insertion of EPM-DN in the leading role of social control of science, especially nursing, and 2017, with the closing of the last decade for the creation of this project, which consolidated the journal as an exponent of scientific dissemination, covering 30 years of research, practically its entire period of existence and strengthening.

### Study setting

APE election was related to the following criteria that exemplify its editorial adherence to quality discourse: (a) current editorials and those started before 1988 on nursing, for demonstrating continuity and persistence of editorial members; (b) not having changed its name throughout its history, in order to maintain the editorial line and persistence in the same title; (c) public, free, digital and complete availability of the entire published collection, by adhering to the requirement of total digitalization of content; (d) large collection published in the Scientific Electronic Library Online (SciELO), as it presents a demanding level of indexing in databases.

The authors were inserted in the institutional context at different levels of proximity to the editorial board: graduate student, professor or editorial committee member. However, none of them participated in the editing of the journal in the established time frame, which demonstrates proximity to the subject matter, as they were in the same institution, but not influential in the editorial team or in the decisions made until then for internal strategies.

### Data source

Inclusion criteria for the study consisted of participants’ role on the editorial board from 1988 to 2017 in one of the following positions: scientific editor and, in the absence of this, editor-in-chief of APE or director of EPE. It is worth noting that there were moments in the history of the journal studied in which the school directors simultaneously took over the role of journal editors, which was the justification for the invitation to participate in the interviews.

### Data collection and organization

After the survey of 12 names indicated for the research, all were invited to participate: two did not respond to the established contacts; two were not in a health condition to collaborate in the research; and one declined to participate. Thus, the possibility of interview was restricted to seven female participants.

Due to the COVID-19 pandemic, which brought social isolation rules to prevent infections, it was decided to offer participants the opportunity to be interviewed using a semi-structured form, via teleconference, using various applications, whose sounds were recorded normally. Only one participant requested an in-person interview, respecting the distance of two meters, use of a mask and sanitizing the environment with 70% alcohol. The interviews were conducted between April 11 and August 10, 2021.

### Work stages

With the oral recording recorded, the following oral source treatment stages were chosen: transcription (written record, detailed and adapted to spoken language) with return to participants, who had the opportunity to extract or correct excerpts, according to negotiation between researcher and participant.

With the transcription approved by participants, we moved on to the textualization phase (text rearrangement to divide textual information according to the research question, chronology or topis), maintaining the meaning of participants’ statements.

Finally, we moved on to transcreation preparation (summary of the interview from the researcher’s perspective, respecting the meaning expressed by participants). This research obtained seven transcreated documents or seven statements.

They were then coded, with thematic elements that converged and diverged from each other being identified. Given the similarity and relevance of codes, they were categorized according to their relationship with the discursive formations presented.

Statements and codes were given to a researcher in nursing history, external to supervision, with a doctoral degree and experience in oral history, who gave his opinion on the correspondence between the audio text and the written text.

### Data analysis

The data were divided into three decades (1988 to 1997; 1998 to 2007; 2008 to 2017), and the following thematic categories were extracted: motivation; management; financing; partnerships; digitalization; indexing; and internationalization. From these categories, a discourse analysis was carried out of statements that could represent forms of social control in publishing, which resulted in three discursive categories presented in this article: knowledge registration and circulation, knowledge internationalization and scientific assessment centrality displacement, respectively, for the first three decades, exemplified by parts of the statements, in order to enrich the results.

The study of discourse was proposed by several philosophers, including the French philosopher Michel Foucault (1926-1984) and his Archaeology of Knowledge, which proposed the description and historical analysis through discursive elements (discourses and knowledge) and/or non-discursive elements (institutions, political and economic practices)^(8)^. The aim was to understand an internal order: how knowledge developed in a given space of time and in a given culture, which varies according to the focus of the research.

To perform a Michel Foucault’s discourse analysis, it is necessary to enter the universe of the unknown. Statements can be described without the need for interpretations or analysis of subjective meanings; it is enough to simply state them in the way they were found^(9)^. To extract statements, it is necessary to: (1) detach oneself from the traditional categories of the history of ideas or literature (author, book, work); (2) extract spoken or written speeches (statements) according to their dispersion and singularity; (3) describe the situations that have or have not promoted the statements^(10)^.

The social control promoted by speeches and their statements can be observed from the perspective of panopticism, a fundamental characteristic of society, where there is individual and continuous surveillance for control, punishment and reward, even if correction is necessary for this, i.e., the formation and transformation of individuals through certain norms^(10)^.

## RESULTS

### First decade

The motivation for creating the journal came from nurses’ academic context and their scientific communication vehicles available at that time, a situation that influenced the professional and social image of nurses in Brazil:

*In the 1980s, few of us had reached the master’s level.* [...] *our concern was to produce increasingly cohesive scientific output. This would only happen if there was a serious space, eminently academic and concerned with scientific and methodological rigor and quality.* (D1)

*UNIFESP did not have a journal, with the exception of APE.* (D5)

*The graduate program gave strength and support to the journal by publishing the results of theses and monographs. A large percentage of these works were from the school itself.* (D6)

*All of these Brazilian journals are linked to nursing schools and were created in the same way: to house the scientific production of graduate programs, since doctorates were born 30 years ago.* [...] *these needed to channel scientific production. We had no Brazilian journals, with the exception of REBEn, an important journal of our association, but insufficient. The ABEn journal could not absorb everything that was produced, so the birthplace of nursing journals was in schools.* (D7)

With the creation of APE within the school’s internal context, the need for partnerships was felt for its growth, a behavior that brought with it discourses of other institutions of great national prestige in scientific production, mainly in technical and editorial aspects:

*The journal’s relations with other universities have been successful, due to the personal proximity with colleagues from the Schools of Nursing of the Universidade de São Paulo - São Paulo and Ribeirão Preto Campuses - responsible for publishing, respectively, the Revista de Enfermagem and Revista Latino-Americana.* (D2)

In this regard, concern with APE’s editorial quality before scientific peers was mixed with the incipient financial obstacles, especially to achieve the first experiences of indexing in databases:

*To index a journal, you need to have a certain weight, maturity, and quality of work, otherwise you won’t get indexed.* (D1)

*My work began due to the lack of regularity caused by financial problems that could lead to the loss of indexing in the LILACS database.* (D2)

From a discursive point of view, the association between the discourse of editorial quality and the need for knowledge recording/circulation was explored, with statements of professional valorization in scientific and academic settings, through the need for scientific production in nursing schools, mainly graduate studies, with incipient regularity and mostly internal to the school.

### Second decade

Editorial maturity, with the changes and learnings of the first decade, brought new institutional interfaces, reinforcing the need to seek publication aligned with the most complex, internationalized and extremely competitive economic system:

*Specifically, the implementation of the Coordination for the Improvement of Higher Education Personnel* [CAPES] *Qualis system, Scientific Electronic Library Online* [SciELO]*, bibliometric indicators and the implementation of digital technologies were fundamental to encouraging qualitative changes* [...]. (D2)

*According to the CAPES assessment, having your own journal counts towards the school’s assessment score.* [...] *at first, it was domestic in nature, but then it began to serve the scientific community.* [...] *I’m also concerned because, from the moment you enter a business, you have to remain competitive. If you’re not competitive, they don’t want you anymore.* [...] *as I said, a journal is a business. Where there’s business, there’s money, there’s payment.* (D4)

*You embrace this world of the health area, develop your practical work, and are recognized by society, but scientifically, what you produce is only recognized when you publish your results in an impactful journal, that is, indexed by an international agency, which is shared with everyone, in this case, in the health area.* (D6)

International discourses brought by the large indexing bases, mainly the private ones, reinforced the editorial environment of global competitiveness, a change in the perception of simply recording and circulating knowledge, in addition to scientific work and peer recognition at levels different from those practiced in the first decade of the journal’s existence:

*There was concern about indexing in different databases, with a view to future partnerships.* (D4)

*For a scientific journal, indexing is fundamental. A journal needs to be indexed by international agencies and respected for the rigor of scientific work in the journal of methods and results.* (D6)

*One challenge is to enter the database; another is to be assessed annually to remain there; it is a huge task.* (D3)

Upon the acceptance of globalization of scientific knowledge, the official discourse related to the scientific production cycle has come closer to the concept of quality and excellence in publishing, especially in the search for the virtualization of articles, made available worldwide:

*We were very concerned about maintaining the quality of our articles.* [...] *there were different levels of quality between articles, especially at the beginning of my term as executive editor. It was very difficult to deal with this because we wanted to give everyone a chance, but it was complicated to correct the articles that needed improvement.* (D3)

*If the best researchers seek out other journals to publish their best results, the journal’s impact factor does not increase, because the world will not cite it. The H factor declines. The journal must always be concerned about this.* (D4)

From a discursive point of view, the association between discourse of editorial quality and the need for knowledge internationalization was also explored, with statements of market vision, editorial panopticism, elevation of article selection criteria to compose an edition that would have international impact, indexing and full digitalization of content.

### Third decade

There was a strengthening of the discourse developed in the first two decades, represented by knowledge registration and circulation, added to globalization of science, still focused on the scientific community, generally limited to its actors and means, such as reviewers, editors, researchers, the digital world, databases of public and private companies, indexing, etc. It is worth highlighting the fact that this decade brings the relationship between the journal and the school of nursing that provides it as an instrument for disseminating scientific production, a non-discursive element, i.e., institutional, which has probably influenced the discursive elements of editorials in APE:

*The graduate program gave strength and support to the journal by publishing the results of theses and monographs. A large percentage of these works were from the school itself.* (D6)

*This is an interesting discussion, because the journal is the official journal of a school, as it is at USP in São Paulo, USP Ribeirão, the nursing school in Porto Alegre, Santa Maria, Bahia, and Minas Gerais. All of these Brazilian journals are linked to the nursing school and were created in the same way: to house the scientific production of graduate programs, since doctorates were born 30 years ago.* (D7)

Another non-discursive element identified was the movement to open up science, in which scientific quality assessment broke the exclusive bubble of editorial members, consolidated for decades in academic environments.

*There is a complicated movement of preprints, of open science, that we need to join. The journal has been trying to do this for the last decade.* [...] *we will give visibility to the review. The author of the article will know who the reviewer is who is reading it, and it will be clear that it is written. This is the open science movement, and we are doing this gradually.* (D7)

However, the effects of mass dissemination to raise bibliometric levels brought APE closer to popular social networks, bringing media discourses outside such traditional and formal scientific communication circuits.

*We are not just talking to people of my generation, but also to young nurses and researchers.* [...] *the journal brings visibility to the school. Opening up to social media was a successful project for the journal.* [...] *it responds very well, it knows how to use social media, with investments in Facebook^®^, Twitter^®^, etc.* [...] *we have roles to follow up on, we need to increase the number of citations of the journal. We will continue to form national and international partnerships to continue on this path.* (D7)

In a discursive manner, there was the insertion of the discursive formation, still incipient and current, of the scientific assessment centrality displacement, with preprint statements, open science, exclusive digitalization, the mediatization of science, whose regularity and interaction with discourses of previous decades were simultaneous and with an interface predominantly external to the school.

## DISCUSSION

The official discourse, or in Michel Foucault’s terms, the known knowledge, stated by the journal itself, described the need for quality criteria^(6)^, with clear and technical information about its editorial characteristics, such as content provision mode (open access), access via mobile device application, institutional support, editorial board qualification, indexing in international and national databases, classification through the CAPES Qualis Journals index, use of a specialized platform for submitting and monitoring manuscripts, selecting original works for publication and enhancing bibliometric indicators, especially citing articles.

In the present research, unofficial discourses or buried knowledge (Michel Foucault) can be summarized in three aspects, such as knowledge registration and circulation, knowledge internationalization, and scientific assessment centrality displacement, combined with each other, which probably manifested themselves since the beginning of the journal, but which were detected through participants’ speeches at different times, according to the study of each decade.

The discourse of scientific quality based on methodological rigor was a point to be taken into consideration by Brazilian nurses when constructing their research pillars. Although nursing did not yet have its own philosophical body, its characteristic as an applied science allowed it to absorb Cartesian, positivist, dialogical and phenomenological determinations. Thus, the first international and even national journals played this preponderant role of professional valorization, an important statement for the discourse of knowledge registration and circulation^(11-14)^.

It is now recognized that graduate studies have driven the enunciation of valorization of nursing professional identity from a scientific perspective. Scientific research is not dissociated from the context of healthcare, the professional class and the defense of social ideas^(15,16)^. Nursing scientists’ political action has strengthened professional identity in society through contemporary science^(17)^. In this regard, it is necessary to reflect on whether the differentiation between nurses and other healthcare professionals was based on the same statements of appreciation as when registration in journals recognized as scientific by society began^(18)^.

The development of nursing science can be positively influenced, from an archaeological point of view, by medical discourses in a natural, consensual and even logical manner. In the Western system, health care has a strong appeal to the study of disease, biology, technology and medical care as a way of understanding health care in general. It is in the hospital that nursing will witness the power of medical discourses in the construction of knowledge and scientific development, with registration in scientific journals. Furthermore, discourses created in hospital settings have driven science to seek social responses through generalizations and predictions. In this context, rationalism is imbued with the social desire to reduce individuals’ distress, i.e., technology would have the answer to all these problems^(19)^. It would be difficult for nursing researchers, and for graduate institutions, not to state the defense of medicalization, technologies, rationalism and capitalism to produce their knowledge and publish it in scientific journals^(17)^.

This need for scientific records to be shared extensively promoted knowledge internationalization as an editorial goal for APE, bringing with it discourses associated with scientific productivity and scientometrics, which helped to understand the formation of scientific researchers’ hierarchical and social logic. In the international scenario, impact factor appreciation was defended as synonymous with the desired quality factor for scientific journals^(20)^. Hence, to raise this bibliometric variable, even the partnership between national and international researchers was defended, whose socialization reinforced the need for this discourse^(21)^.

Not producing Brazilian knowledge in English has become synonymous with scientific isolation, considered as opposition to the international discourse of the construction of science, which defined a flow of adaptation, acculturation and adaptation to the parameters of external rules, naturally stated within the editorials of international scientific journals^(22)^.

This discourse reached Brazilian science policies. Internationalization valued the growth of scientific and academic knowledge, which reinforced globalization of science as something to be achieved^(23)^. Thus, the government took measures to encourage this international expansion, with the discourse of scientific and technological construction placing higher education institutions as the main actors in this execution^(24)^.

In this context, it was impossible to dissociate the internationalization of scientific journals from the need for costs that could make this strategy a reality. National research funding agencies applied such strict criteria to scientific publishing that financial incentives did not tend to reach editorial lines with little adherence to internationalization. Thus, these agencies created discourses that parameterized the ideals of scientific dissemination, which included and excluded publications from these financial incentives^(25)^.

The Brazilian version of globalization of science did not adequately consider the fact that international research funding agencies included publication costs in scientific journals when projects had previously been approved. The same did not occur extensively in national research funding agencies. Thus, the discourse of internationalization was left to authors and publishers who wished to publish articles and books worldwide^(26,27)^.

In addition to these editorial contexts, nursing has been influenced by other aspects of this internationalization discourse, including ontological ones, through other foreign sources of information, exemplified by international manuals of accreditation, diagnoses and nursing interventions, increasingly adopted in their training and work environments, in addition to academic exchanges and partnerships with foreign research institutions^(28)^.

It was understood that science produced and disseminated only regionally would be a conceptual error of scientific work, with a view to the mandatory knowledge internationalization produced in a given regional and cultural context^(22)^. This discursive adherence to internationalization brought different values, criteria and points of view experienced by authors, reviewers and editors in a national context, a paradox that could bring about the denial or acculturation of strictly international models, a movement that can be characterized as the opposite discourse: the “foreignization” of national scientific journals^(26)^.

Thus, the discursive model focused on local social needs caused constant discursive friction with journals, already praised for their impact factor and with successful experiences in the internationalization of their articles. Funding agencies from countries such as Finland, Portugal, Germany, Canada, the United Kingdom, the United States, Poland, India, Belgium, China, Korea, France, the Netherlands, Japan, Norway and Austria sought recognition for their research not only in bibliometric indexes, but for the importance of research within their societies. Seen in these terms, open science enunciation brought the perspective of looking at scientific production for social needs: what was invested in academia needed to be reversed into concrete benefits for the well-being of people in their surroundings, who should receive all the advantages of the construction of new knowledge^(25)^.

This discursive clash of dominance of science promotion for scientific journals versus social need, called in the present research a change in scientific assessment centrality, presented itself more as a discursive formation than a perfectly identifiable discourse in the third decade, an archaeological phenomenon that is still very challenging for theoretical analysis, due to its contemporaneity and the need to observe non-discursive elements.

It must be accepted that scientific research and editorial production are forms of social organization and that, in order to maintain their entropy, evolution and expansion achievements, rules and norms are necessary. Therefore, surveillance within and outside APE has become a necessity. Since modernity, monitoring and controlling social activities has ceased to be an exclusive responsibility of religious institutions or the State, as private institutions have also entered this process of discursive enunciation^(29)^. It is worth noting that bibliometric indexes of great importance for journals from public institutions, such as impact factor and H-index, were first outlined by privately funded databases that sold this type of bibliometrics.

Immersed in this theoretical line, scientific and editorial societies can be observed by their non-discursive elements, i.e., by people, groups, institutions and companies that observe their members in terms of maintaining control over scientific work, a social process called panopticism.

The panoptic structure focuses on scientific and editorial regulation, and APE members have been monitored by external social elements throughout their entire existence and, in more recent times, due to their international achievements, becoming the center of this structure for other social sectors subordinate to their influence. When an individual, group or institution reaches the center of this structure, they are able to control the actions of their subordinates, observing excesses that could disturb an expected order^(29)^.

In the third decade of APE, in addition to the academic and non-academic social sectors identified until then for social control of science, the inclusion of the leading role of authors and readers was noted - of the statements of financing, open science, social media, still incipient - which probably continues into the present time. Thus, social control, built until then by a vertical and hierarchical structuring of the social order (publishing versus reader/author), also brought about the horizontal ordering of economic contours (urban infrastructure, institution, family)^(30)^.

If previously the discursive dialogue was constructed between mostly editorial elements, concerned with the indexing and classification of articles according to H-index and impact factor, originating from traditional circles (from commercial but scientific indexing bases), new non-editorial social sectors, such as Google Scholar^®^, began to dispute centrality in the panoptic structure of scientific communication. Thus, bibliometric indexes began to be multiple, depending on their source, editorial or not, scientific or not, whose logic included downloads, shares on social networks and likes, with all these performance indexes consolidated in social media and streaming services^(31,32)^.

Open science enunciation began to empower authors and readers and also to monitor and punish scientific research, breaking the exclusive bubble of scientific reviewers. Panopticism is a type of power exercised in the form of individual and continuous surveillance that allows control, punishment and reward in the form of corrections. In this context, people transformed themselves as they adapted to the established rules and, consequently, modified the panoptic structure^(33)^.

If APE needed to directly discipline publishing through scientific opinions and editors, in line with the demands of traditional external institutions, confrontation/partnership of statements from social media began, which included, in this disciplinary order, any individual interested in reading research, even if not part of the traditional group of editorial elements^(34,35)^. Social media presents such important statements in society that they have the power to increase bibliographic citation, i.e., social networks positively influence bibliometric indexes, an effect so desired by editorial teams^(31)^.

However, these social networks, which are fundamentally commercial, are not limited to scientific dissemination tools; they bring with them the fight for editorial and scientific quality centrality, an ongoing panoptic crisis, with an impact on the construction of a disciplinary society linked to standardization procedures, whose issues are both social and economic^(33)^. As a reaction to this movement of panoptic centrality displacement, a specialized medical journal proposed the creation of a position of social media editor, who would have the role of observing and monitoring the dynamics of making articles available on popular and commercial platforms, a fact that demonstrated the incipient approximation of media discourses to journal editorials, an indication of panoptic centrality displacement^(36)^.

### Study limitations

The data collected can be discussed with other editorials published in current national nursing journals in the same time frame to triangulate the statements, reinforcing, refuting, or adding new discourses to the context presented. Furthermore, a more in-depth analysis of non-discursive elements, such as people and institutions from the first and second decades, could strengthen the study of a more complete discursive formation, which is complex to present in just a few pages of an article, deserving a dissertation or doctoral thesis.

### Contributions to nursing, health or public policy

This research brought more discursive and qualitative elements to understand the dynamics of the creation, strengthening and maintenance of a nursing journal, whose results went beyond technical, editorial, scientific and bibliometric issues, encompassing sociological views of the work of an editorial team and its response to social demands, with more externalist views of scientific communication.

## FINAL CONSIDERATIONS

APE’s editorial discourses from 1988 to 2017 were based on editorial quality according scientific knowledge social demands. It was understood that a dynamic and changing society would demand changes in its path, with the addition of new and increasingly demanding discourses, which demanded new ways of looking at scientific communication from editors, reviewers, school professors, graduate students and the scientific community.

From the need to enunciate the flow of internal scientific production of undergraduate and graduate courses at EPE, the requirement for international publication with relevant research and, recently, the impact of social media in disseminating its contents, an environment composed of several confluent, antagonistic and complementary discursive elements was perceived.

The social control of scientific research and communication is a difficult topic to discuss, as this analysis works with the power that permeates sociological relations among members of the same group, exposing the differences in social gains embedded in scientific work, which change over time and, to maintain them, clashes are necessary that are silenced in the group through discourses that must be defended and exalted.

In light of this scenario, APE had to adapt to form its official discourse, which enabled it, over the years, to change its initial peripheral position to a central one within scientific communication, supporting its panoptic role. The panopticism developed by APE’s editorial team demonstrated its struggle to be closer to the center of scientific production surveillance. Despite still being on the periphery of international decisions, where it fulfills its role of receiving and adapting to the various discourses of North American and European countries, in the national context, it belongs to an elite group of nursing and health journals that positions itself as a reference for the necessary editorial changes, as if it were one of the spokespeople for international journals, bringing a power of centralization that demands a lot of energy to maintain its entropy, represented by statements that circulate under its surveillance. From Michael Foucault’s point of view, it is a positivity that deserves to be highlighted in studies on nursing journals in Brazil.
