# Supplementary material for: Editorial discourses in the history of Acta Paulista de Enfermagem (1988-2017)
Source: Rev Bras Enferm. 2024 Dec 16;77(6):e20230362. doi: 10.1590/0034-7167-2023-0362 (PMC11654550; doi:10.1590/0034-7167-2023-0362)
Supplement: Translation

# Translation: Discursos editoriais na história da Acta Paulista de Enfermagem (1988-2017)

**DOI:** 10.1590/0034-7167-2023-0362pt

**Authors:** Ricardo Quintão Vieira, Alexandre Pazetto Balsanelli, Vanessa Ribeiro Neves

**Affiliations:** I Universidade Federal de São Paulo. São Paulo, São Paulo, Brasil

**Keywords:** História da Enfermagem, Publicação Periódica, Discurso, Editoração, Controles Informais da Sociedade.

## Abstract

**Objetivos:**

analisar os discursos editoriais da Acta Paulista de Enfermagem de 1988 a 2017.

**Métodos:**

pesquisa qualitativa, histórica, oral, com entrevista com editores da revista. Os depoimentos foram categorizados e apresentados em três decênios, discutidos sob a ótica arqueológica de Foucault.

**Resultados:**

sete depoimentos trouxeram três discursos. No primeiro decênio, o discurso de registro e a circulação do conhecimento apresentaram enunciados de pós-graduação e reconhecimento profissional. Somou-se ao segundo decênio a internacionalização do conhecimento, com enunciados de negócio e panoptismo editoriais, critérios de seleção, indexações e digitalização. Por último, acrescentou-se o discurso de deslocamento da centralidade da avaliação científica com enunciados de *preprint*, ciência aberta, digitalização exclusiva e midiatização da ciência.

**Considerações Finais:**

a revista precisou se adequar para formar o seu discurso oficial, o que possibilitou, ao longo dos anos, mudar sua posição periférica inicial para a central dentro da comunicação científica, corroborando o seu papel panóptico.

## INTRODUÇÃO

Os periódicos científicos são os principais pilares da divulgação da ciência moderna, devido ao poder de disseminar o conhecimento e promover o desenvolvimento da literatura científica de modo exponencial e rápido^(1)^. Na área da enfermagem, esses recursos comunicacionais são atualmente os formatos de documentos mais citados na literatura internacional^(2)^.

Historicamente, *The American Journal of Nursing* é considerado o primeiro periódico mundial de enfermagem nos modelos vigentes de editoração e impressão em papel, tendo sido criado em 1900, nos Estados Unidos da América^(3)^. Por sua vez, a Revista *Annaes* de Enfermagem representou uma experiência editorial brasileira desde a década de 1930, e hoje é conhecida como Revista Brasileira de Enfermagem (REBEn), sendo o periódico de enfermagem vigente mais antigo no contexto nacional.

Logo após a criação da Escola de Enfermeiras do Departamento Nacional de Saúde Pública, em 1923, hoje denominada de Escola de Enfermagem Anna Nery, no Rio de Janeiro, as enfermeiras norte-americanas incutiram, nas enfermeiras profissionais novatas brasileiras, a ideia de que uma revista progrediria a profissão^(4)^.

Esse periódico histórico manteve uma posição exclusiva como veículo de divulgação de instituições acadêmicas até o ano de 1967, quando a Universidade de São Paulo lançou o primeiro número da Revista da Escola de Enfermagem da USP (REEUSP), seguida, anos depois, da Revista Gaúcha de Enfermagem (RGE), da Universidade Federal do Rio Grande do Sul, em 1976.

Na década de 1980, foram lançados dois periódicos de enfermagem: na Universidade Federal da Bahia, em 1981, com o título de Revista Baiana de Enfermagem (RBE), seguida, em 1988, da Acta Paulista de Enfermagem (APE), pelo Departamento de Enfermagem da Escola Paulista de Medicina (DE-EPM), hoje Escola Paulista de Enfermagem (EPE) da Universidade Federal de São Paulo (UNIFESP), o que destacou o fato de haver, desde então, duas revistas de enfermagem na capital paulista.

O objetivo da APE é publicar resultados de pesquisas e melhorar as práticas de cuidado nos diferentes contextos de atenção à saúde, fazendo parte do Regimento Interno da EPE^(5)^. Durante o seu crescimento e amadurecimento como veículo de comunicação científica da enfermagem ao longo das décadas, a APE desenvolveu um discurso atual baseado em critérios de qualidade e metas editoriais para a busca da excelência em divulgação científica, tais como indexação em bases de acesso global (PubMed Central e MEDLINE), alcance da estratificação A1 no Qualis da Coordenação de Aperfeiçoamento de Pessoal de Nível Superior (CAPES), aumento do fator de impacto, “Publicação Contínua”, consolidação do Portal RevEnf e adesão ao *preprint* ^(6)^.

Os discursos apresentados pela APE, e não apenas por essa revista, mas por todos os editores de periódicos que buscam e se esforçam para alcançar e manter os critérios de qualidade, representam, de forma sociológica, a manutenção das normas e das regras da ciência como um todo.

O discurso é toda atividade comunicativa que traz sentido entre interlocutores nas suas relações interacionais. Para se pensar em discurso, é necessário entender que há um lugar social onde o discurso é construído, por quem é dito, a quem se diz e sua convivência com outros discursos que circulam socialmente^(7)^. Sob essa ótica, a história oficial, em especial do discurso oficial, traz elementos importantes para o entendimento da trajetória da APE como veículo de informação científica, porém apresenta limitações para a discussão teórica e sociológica, fato que empobrece a profundidade das formações discursivas que estão ocultas entre os seus membros.

Diante desse quadro teórico, e aceitando a premissa de que os periódicos, em especial os editores científicos, participam da comunicação científica e são influenciados pelas forças que sustentam os controles social e científico da literatura de enfermagem, questionou-se como se deram os discursos editoriais pela APE do ano de 1988 a 2017.

## OBJETIVOS

Analisar os discursos editoriais desenvolvidos pela APE de 1988 a 2017.

## MÉTODOS

### Aspectos éticos

O projeto foi submetido à apreciação do Comitê de Ética em Pesquisa da UNIFESP, conforme as recomendações da Resolução do Conselho Nacional de Saúde n^o^ 466/12 do Ministério da Saúde, com aprovação da condução da pesquisa em agosto de 2019. Para a coleta de dados, foi solicitada a anuência da participante por meio do Termo de Consentimento Livre e Esclarecido, obedecendo-se às recomendações do Ofício Circular n^o^ 2/2021/CONEP/SECNS/MS, de 24 de abril de 2021, sob o título “Orientações para procedimentos em pesquisas com qualquer etapa em ambiente virtual”.

### Tipo de estudo

Estudo histórico, que consiste em observar e descrever qualitativamente a relação social estabelecida entre o homem e o seu meio, cujos efeitos podem ser observados no presente momento, sob a gama complexa de visões, abordagens e perspectivas. No presente estudo, optou-se pela história oral, baseada em fontes orais registradas e tratadas. Além disso, essas fontes foram analisadas à luz da Arqueologia do Saber, focada em formações discursivas (elementos discursivos e não discursivos).

Para atender ao processo de reprodutibilidade, a presente pesquisa pode ser resumida nas seguintes etapas: levantamento histórico do objeto de estudo; recorte temporal justificado; eleição do cenário de pesquisa; participantes (com critérios de inclusão e exclusão); agendamento das entrevistas; gravação em áudio (ou vídeo) por meio de questionário semiestruturado; conversão da fala oral para a fala escrita (transcrição, textualização, transcriação); codificação; categorização; apresentação dos resultados com análise do discurso (formações discursivas “soterradas”); discussão dos resultados com a literatura atualizada conforme os elementos discursivos “desenterrados” (expostos).

Utilizou-se o *checklist* proposto pela *COnsolidated criteria for REporting Qualitative research* (COREQ): *a 32-item checklist for interviews and focus groups*, em todos os seus domínios: *research team and reflexivity; study design; data analysis*.

### Procedimentos metodológicos

O recorte temporal compreendeu o intervalo entre 1988, ano em que ocorreu a primeira publicação da APE, marco da inserção do DE-EPM no protagonismo do controle social da ciência, em especial da enfermagem, e o ano de 2017, com o fechamento do último decênio para a confecção deste projeto, o que consolidou a revista como expoente de divulgação científica, cobrindo 30 anos de investigação, praticamente todo o seu período de existência e fortalecimento.

### Cenário de estudo

A eleição da APE relacionou-se aos seguintes critérios que exemplificam a sua adesão editorial ao discurso da qualidade: (a) editoriais correntes e iniciados antes de 1988 de enfermagem, por demonstrar continuidade e persistência dos membros editoriais; (b) não ter mudado de denominação ao longo de sua trajetória, para manter a linha editorial e a persistência no mesmo título; (c) disponibilização pública, gratuita, digital e integral de toda a coleção publicada, por aderir à exigência de digitalização total de conteúdo; (d) grande coleção publicada na *Scientific Electronic Library Online* (SciELO), por esta apresentar um exigente nível de indexação em base de dados.

Os autores estavam inseridos no contexto institucional em diversos níveis de aproximação com o corpo editorial: estudante de pós-graduação, docente ou membro do comitê editorial. No entanto, nenhum deles participou da editoração do periódico no recorte temporal estabelecido, o que demonstra aproximação com o objeto do assunto, por estar na mesma instituição, porém não influente na equipe editorial ou nas decisões até então tomadas para as estratégias internas.

### Fonte de dados

Os critérios de inclusão na pesquisa consistiram na atuação da participante na comissão editorial, de 1988 a 2017, em um dos seguintes postos: editor científico e, na ausência deste, o editor-chefe da APE ou o diretor da EPE. Destaca-se que houve momentos na história do periódico estudado nos quais os diretores da escola assumiram concomitantemente a função de editores do periódico, justificativa para o convite de participação nas entrevistas.

### Coleta e organização dos dados

Após o levantamento de 12 nomes indicados para a pesquisa, todos foram convidados para a pesquisa: dois não responderam aos contatos estabelecidos; dois não estavam em condições de saúde para colaborar na pesquisa; e um declinou da participação. Assim, a possibilidade de entrevista restringiu-se a sete participantes do sexo feminino.

Devido à pandemia de COVID-19, que trouxe regras de isolamento social para prevenção de infecções, optou-se por oferecer às participantes a oportunidade de entrevista com formulário semiestruturado, via teleconferência, por diversos aplicativos, cujos sons foram gravados normalmente. Somente uma participante solicitou a entrevista no modelo presencial, respeitando-se o distanciamento de dois metros, uso de máscara e higienização do ambiente com álcool 70%. As entrevistas foram realizadas entre 11 de abril e 10 de agosto de 2021.

### Etapas do trabalho

Com o registro oral gravado, optou-se pelas seguintes etapas de tratamento da fonte oral: transcrição (registro escrito, detalhado e adaptado à linguagem falada) com devolução aos participantes, que tiveram a oportunidade de extrair ou corrigir trechos, conforme a negociação entre pesquisador e participante.

Com a transcrição aprovada pela participante, passou-se para a fase de textualização (rearranjo do texto para se dividir as informações textuais conforme a pergunta de pesquisa, cronologia ou temática), com a manutenção dos sentidos das falas das participantes.

Por último, passou-se para a confecção da transcriação (síntese da entrevista sob a perspectiva do pesquisador, respeitando-se o sentido dito pela participante). A presente pesquisa obteve sete documentos transcriados ou sete depoimentos.

Posteriormente, eles passaram pelo processo de codificação, com apontamento de elementos temáticos convergentes e divergentes entre si. Diante da similaridade e pertinência dos códigos, promoveu-se a categorização conforme a relação com as formações discursivas expostas.

Os depoimentos e códigos foram entregues a um pesquisador em história da enfermagem, externo à orientação, com titulação de doutor, e com experiência em história oral, que deu seu parecer sobre a correspondência entre o texto de áudio e o texto escrito.

### Análise dos dados

Os dados foram divididos por três decênios (1988 a 1997; 1998 a 2007; 2008 a 2017), e foram extraídas as seguintes categorias temáticas: motivação; gestão; financiamento; parcerias; digitalização; indexação; e internacionalização. Dessas categorias, realizou-se uma análise de discurso de falas que pudessem representar formas de controle social em editoração que resultou em três categorias discursivas apresentadas neste artigo: registro e circulação do conhecimento, internacionalização do conhecimento e deslocamento da centralidade de avaliação científica, respectivamente, para os três primeiros decênios, exemplificados por partes dos depoimentos, a fim de enriquecer os resultados.

O estudo do discurso foi proposto por diversos filósofos, entre eles o francês Michel Foucault (1926-1984) e sua Arqueologia do Saber, que propôs a descrição e análise histórica por meio de elementos discursivos (discursos e saberes) e/ou não discursivos (instituições, práticas políticas e econômicas)^(8)^. Buscou-se entender uma ordem interna: como o saber se desenvolveu em um espaço de tempo e em uma determinada cultura, que varia conforme o foco da pesquisa.

Para se realizar uma análise de discurso foucaultiano, torna-se necessário adentrar o universo do desconhecido. Os enunciados podem ser descritos sem a necessidade de interpretações ou análise de sentidos subjetivos; basta apenas enunciá-los da maneira como foram encontrados^(9)^. Para se extrair enunciados, é necessário: (1) desprender-se das categorias tradicionais da história das ideias ou literatura (autor, livro, obra); (2) extrair falas (enunciados) ditas ou escritas de acordo com a sua dispersão e singularidade; (3) descrever as situações que tenham promovido ou não os enunciados^(10)^.

O controle social promovido pelos discursos e seus enunciados pode ser observado pela ótica do panoptismo, uma característica fundamental da sociedade, onde há uma vigilância individual e contínua por controle, castigo e recompensa, nem que para isso seja necessária a correção, ou seja, a formação e a transformação dos indivíduos por meio de certas normas^(10)^.

## RESULTADOS

### Primeiro decênio

A motivação para a criação da revista deu-se no contexto acadêmico dos enfermeiros e de seus veículos de comunicação científica disponíveis naquele momento, situação que influenciava a imagem profissional e social do enfermeiro no Brasil:

*Na década de 1980, poucas de nós tinham atingido o nível de mestrado.* [...] *a nossa preocupação era fazer uma produção cada vez mais coesa no aspecto científico. Isso somente ocorreria se houvesse um espaço sério, eminentemente acadêmico e preocupado com a rigidez e qualidade científica e metodológica.* (D1)

*A UNIFESP não tinha revista, com exceção da APE.* (D5)

*A pós-graduação deu força e subsídio para a revista com a publicação de resultados de teses e monografias. Grande percentual desses trabalhos era da própria escola.* (D6)

*Todas essas revistas brasileiras estão ligadas à escola de enfermagem e nasceram do mesmo jeito: abrigar a produção científica dos programas de pós-graduação, pois 30 anos atrás nasciam os doutorados.* [...] *estes precisavam escoar a produção científica. Não tínhamos periódicos brasileiros, com exceção da REBEN, importante revista da nossa associação, mas insuficiente. A revista da ABEn não dava conta de absorver tudo que era produzido, então o nascedouro das revistas de enfermagem foi nas escolas.* (D7)

Com a criação da APE no contexto interno da escola, sentiu-se a necessidade de parcerias para o seu crescimento, comportamento que trouxe para si os discursos de outras instituições de grande prestígio nacional na produção científica, principalmente nos aspectos técnicos e editoriais:

*As relações da revista com as outras universidades tiveram bons êxitos, devido à proximidade pessoal com as colegas das Escolas de Enfermagem da Universidade de São Paulo - Campi São Paulo e de Ribeirão Preto - responsáveis pela edição, respectivamente, da Revista de Enfermagem e Revista Latino-Americana.* (D2)

Neste aspecto, a preocupação com a qualidade editorial da APE diante dos pares científicos mesclou-se com os incipientes obstáculos financeiros, em especial para conseguir as primeiras experiências de indexação em bases de dados:

*Para indexar uma revista, você precisa ter um peso, uma maturidade, uma qualidade de trabalho, senão você não consegue essa indexação.* (D1)

*Meu trabalho iniciou a partir da falta de regularidade ocasionada pelos problemas financeiros que poderiam conduzir à perda da indexação na base LILACS.* (D2)

Do ponto de vista discursivo, escavou-se a associação entre o discurso da qualidade editorial e a necessidade de registro/circulação de conhecimento, com enunciados de valorização profissional no meio científico e acadêmico, por meio da necessidade de produção científica das escolas de enfermagem, principalmente da pós-graduação, com regularidade incipiente e majoritariamente interna à escola.

### Segundo decênio

A maturidade editorial, com as mudanças e aprendizados do primeiro decênio, trouxe novas interfaces institucionais, reforçando a necessidade da busca pela publicação alinhada ao sistema econômico mais complexo, internacionalizado e extremamente concorrido:

*Especificamente pela implantação do sistema Qualis da Coordenação de Aperfeiçoamento de Pessoal de Nível Superior* [CAPES], *Scientific Electronic Library Online* [SciELO], *indicadores bibliométricos e implementação das tecnologias digitais foram fundamentais para estimular as mudanças qualitativas* [...]. (D2)

*Segundo a avaliação da CAPES, ter uma revista própria conta na pontuação da avaliação da escola.* [...] *a princípio, ela apresentava caráter doméstico, depois passou a servir a comunidade científica*. [...] *também tenho preocupação porque, a partir do momento em que você entra em um negócio, você tem que manter a competitividade. Se você não é competitivo, eles não o querem mais.* [...] *como eu disse, revista é negócio. Onde tem negócio, tem dinheiro, tem pagamento.* (D4)

*Você abraça esse mundo da área da saúde, desenvolve seu trabalho prático, é reconhecido pela sociedade, mas, cientificamente, o que produz só é reconhecido quando publica seus resultados numa revista de impacto, ou seja, indexada em agência internacional, que é compartilhada com todos, no caso, da área da saúde.* (D6)

Os discursos internacionais trazidos pelas grandes bases indexadoras, principalmente as privadas, reforçaram o ambiente editorial de competividade global, uma mudança na percepção do simples registrar e circular o conhecimento, além do fazer científico e do reconhecimento por pares em patamares diferentes daqueles praticados na primeira década de existência da revista:

*Houve preocupação na indexação em diferentes bases de dados, enxergando as futuras parcerias.* (D4)

*Para uma revista científica, as indexações são fundamentais. Uma revista precisa ser indexada por agências internacionais e respeitadas pelo rigor dos trabalhos científicos na revista dos métodos e resultados.* (D6)

*Um desafio é entrar na base; outro é ser avaliada anualmente para a permanência nela; é um trabalho imenso.* (D3)

Com a aceitação da globalização do conhecimento científico, o discurso oficial relacionado ao ciclo da produção científica aproximou-se mais do conceito de qualidade e excelência de editoração, especialmente na busca pela virtualização de artigos, disponibilizados de forma mundial:

*A nossa preocupação em manter o nível dos artigos era imensa*. [...] *havia níveis diferentes entre artigos, principalmente no começo da minha gestão como editora-executiva, era bem difícil lidar porque queríamos dar vez para todo mundo, mas era complicado corrigir os artigos que precisavam ser melhorados.* (D3)

*Se os melhores pesquisadores buscam outros periódicos para publicar seus melhores resultados, o fator de impacto da revista não sobe, porque o mundo não vai citá-la. O fator H declina. A revista tem que ter essa preocupação sempre.* (D4)

Do ponto de vista discursivo, escavou-se também a associação entre o discurso da qualidade editorial e a necessidade de internacionalização do conhecimento, com enunciados de visão mercadológica, panoptismo editorial, elevação dos critérios de seleção de artigos para compor uma edição que tivesse impacto internacional, indexações e digitalização integral de conteúdo.

### Terceiro decênio

Houve o fortalecimento do discurso desenvolvido nas duas primeiras décadas, representadas pelo registro e circulação do conhecimento, acrescido da globalização da ciência, ainda focalizada na comunidade científica, geralmente circunscrita a seus atores e meios, como pareceristas, editores, pesquisadores, mundo digital, bases de dados de empresas públicas e privadas, indexações, etc. Destaque-se o fato de esse decênio trazer as relações entre a revista e a escola de enfermagem provedora como instrumento de divulgação da produção científica, um elemento não discursivo, ou seja, institucional, que provavelmente vem influenciando os elementos discursivos dos editoriais na APE:

*A pós-graduação deu força e subsídio para a revista com a publicação de resultados de teses e monografias. Grande percentual desses trabalhos era da própria escola.* (D6)

*Essa é uma discussão interessante, porque a revista é oficial de uma escola, como é na USP de São Paulo, da USP Ribeirão, da escola de enfermagem de Porto Alegre, de Santa Maria, da baiana, da mineira. Todas essas revistas brasileiras estão ligadas à escola de enfermagem e nasceram do mesmo jeito: abrigar a produção científica dos programas de pós-graduação, pois 30 anos atrás nasciam os doutorados.* (D7)

Outro elemento não discursivo identificado foi o movimento de abertura da ciência, no qual a avaliação da qualidade científica rompeu a bolha exclusiva dos membros editoriais, consolidada há décadas no meio acadêmico.

*Há um movimento complicado do preprint, da ciência aberta, que precisamos aderir. Essa última década a revista está tentando fazer isso.* [...] *vamos dar visibilidade para o parecer. O autor do artigo vai saber quem é o parecerista que está lendo, que aparece que está escrito. Esse é o movimento da ciência aberta, estamos fazendo isso gradualmente.* (D7)

No entanto, os efeitos da divulgação em massa para elevar os níveis bibliométricos aproximaram a APE às redes sociais populares, trazendo discursos midiáticos fora de tais circuitos tradicionais e formais de comunicação científica.

*Não estamos falando apenas com pessoas da minha geração, mas falando com jovens enfermeiros e pesquisadores.* [...] *a revista traz visibilidade para a escola. A abertura para as mídias sociais, isso foi um projeto de sucesso da revista.* [...] *ela responde muito bem, soube usar as mídias sociais, com investimento em Facebook^®^, Twitter^®^, etc.* [...] *temos papéis para dar seguimento, precisamos aumentar as citações da revista. Vamos continuar fazendo parcerias nacionais e internacionais para continuar nesse caminho.* (D7)

De modo discursivo, houve a inserção da formação discursiva, ainda incipiente e atual, do deslocamento da centralidade de avaliação científica, com enunciados *preprint*, da ciência aberta, da digitalização exclusiva, da midiatização da ciência, cuja regularidade e interação com os discursos das décadas anteriores foram simultâneas e de interface predominantemente externa à escola.

## DISCUSSÃO

O discurso oficial, ou de forma foucaultiana, o saber conhecido, dito pela própria revista, descreveu a necessidade de critérios de qualidade^(6)^, com informações claras e técnicas sobre as suas características editoriais, tais como modo de disponibilização de conteúdo (*open access*), acesso em aplicativo de dispositivo móvel, apoio institucional, qualificação de corpo editorial, indexação em bases de dados internacionais e nacionais, classificação por meio do índice Qualis Periódicos da CAPES, utilização de plataforma especializada para a submissão e acompanhamento de manuscritos, seleção de trabalhos originais para a publicação e valorização dos indicadores bibliométricos, em especial da citação de artigos.

Na presente pesquisa, os discursos não oficiais ou os saberes soterrados (foucaultiano) podem ser resumidos em três aspectos, como registro e circulação do conhecimento, internacionalização do conhecimento, e deslocamento da centralidade de avaliação científica, combinados entre si, que provavelmente se manifestaram desde o início do periódico, mas que foram detectados por meio das falas das participantes em momentos diferentes, conforme o estudo de cada decênio.

O discurso da qualidade da ciência baseado no rigor metodológico foi um ponto a ser levado em consideração pelos enfermeiros brasileiros ao construírem seus pilares de investigação. Embora a enfermagem ainda não tivesse um corpo filosófico próprio, sua característica como ciência aplicada lhe permitiu absorver determinações cartesianas, positivistas, dialógicas e fenomenológicas. Desse modo, os primeiros periódicos internacionais e até mesmo nacionais tiveram esse papel preponderante de valorização profissional, um enunciado importante para o discurso de registro e circulação do conhecimento^(11-14)^.

Reconhece-se hoje que as pós-graduações impulsionaram a enunciação da valorização da identidade profissional de enfermagem pelo viés científico. A pesquisa científica não está dissociada do contexto de cuidados de saúde, da classe profissional e da defesa de ideias sociais^(15,16)^. A atuação política dos cientistas de enfermagem fortaleceu a identidade profissional na sociedade por meio da ciência contemporânea^(17)^. Nesse aspecto, é necessário refletir se a diferenciação entre os enfermeiros e outros profissionais de saúde baseou-se nos mesmos enunciados de valorização de quando se iniciou o registro em periódicos reconhecidos como científicos pela sociedade^(18)^.

O desenvolvimento da ciência na enfermagem pode ser influenciado positivamente, do ponto de vista arqueológico, pelos discursos médicos de maneira natural, consensual e até lógica. No sistema ocidental, a atenção em saúde tem forte apelo aos estudos da doença, biologia, tecnologia e assistência médica como forma de entendimento do cuidado da saúde em geral. É no hospital que a enfermagem assistirá ao poder dos discursos médicos na construção do conhecimento e do desenvolvimento científico, com o registro em periódicos científicos. Além disso, são os discursos criados nos ambientes hospitalares que impulsionaram a ciência em buscar as suas respostas sociais, por meio de generalizações e previsões. Nesse contexto, o racionalismo está impregnado do desejo social de diminuir os sofrimentos dos indivíduos, ou seja, a tecnologia teria a resposta para todos esses problemas^(19)^. Seria difícil para o pesquisador de enfermagem e para as instituições de pós-graduação não enunciarem a defesa da medicalização, de tecnologias, do racionalismo e do capitalismo para produzir seus conhecimentos e publicá-los em periódicos científicos^(17)^.

Essa necessidade de registro científico a ser compartilhado de forma extensiva promoveu a internacionalização do conhecimento como meta editorial para a APE, trazendo para si os discursos associados à produtividade científica e à cienciometria, que ajudaram a compreender a formação da lógica hierárquica e social dos pesquisadores científicos. No cenário internacional, defendeu-se a valorização do fator de impacto como sinônimo de fator de qualidade desejado para os periódicos científicos^(20)^. Desse modo, e para elevar essa variável bibliométrica, defendeu-se até mesmo a parceria entre pesquisadores nacionais e internacionais, cuja socialização reforçou a necessidade desse discurso^(21)^.

Não produzir o conhecimento brasileiro em língua inglesa tornou-se o sinônimo de isolamento científico, considerado como oposição ao discurso internacional da construção da ciência, que definiu um fluxo de adaptação, aculturação e adequação ao parâmetro de regras externas, naturalmente enunciadas dentro dos editoriais de revistas científicas internacionais^(22)^.

Esse discurso chegou às políticas científicas do Brasil. A internacionalização valorizou o crescimento do conhecimento científico e acadêmico, o que reforçou a globalização da ciência como algo a ser conquistado^(23)^. Desse modo, o governo tomou medidas de incentivo a essa expansão internacional, com o discurso da construção científica e tecnológica posicionando as instituições educacionais de nível superior como atores principais dessa execução^(24)^.

Nesse contexto, era impossível dissociar a internacionalização dos periódicos científicos da necessidade de custos que pudessem concretizar essa estratégia. As agências nacionais de fomento de pesquisa aplicaram critérios tão estritos de editoração científica que o incentivo financeiro não tendia a chegar em linhas editoriais com pouca aderência à internacionalização. Assim, esses órgãos criaram discursos que parametrizaram os ideais da divulgação científica, que incluíam e excluíam publicações desses incentivos financeiros^(25)^.

A versão brasileira da globalização da ciência não considerou de maneira apropriada o fato de que as agências internacionais de fomento à pesquisa incluíam os custos de publicação em periódicos científicos quando os projetos estavam anteriormente aprovados. O mesmo não ocorreu de forma extensiva nos órgãos nacionais de incentivo à pesquisa. Dessa forma, o discurso da internacionalização ficou a cargo de autores e editores que desejassem publicar artigos e livros para todo o mundo^(26,27)^.

Além desses contextos editoriais, a enfermagem sofreu outras influências desse discurso de internacionalização, inclusive o ontológico, por meio de outras fontes de informação estrangeiras, exemplificadas pelos manuais internacionais de acreditação, de diagnósticos e de intervenções de enfermagem, cada vez mais adotados em seus ambientes de formação e de trabalho, além dos intercâmbios acadêmicos e das parcerias com instituições estrangeiras de pesquisa^(28)^.

Entendeu-se que a ciência produzida e divulgada apenas de forma regional seria um erro conceitual do trabalho científico, com vistas à internacionalização obrigatória dos conhecimentos produzidos em determinado contexto regional e cultural^(22)^. Essa adesão discursiva à internacionalização trouxe valores, critérios e pontos de vista diferentes vividos por autores, revisores e editores em contexto nacional, paradoxo que poderia trazer a negação ou a aculturação de modelos estritamente internacionais, movimento que pode ser caracterizado como o discurso oposto: a “estrangeirização” do periódico científico nacional^(26)^.

Assim, o modelo discursivo voltado para as necessidades sociais locais provocou atritos discursivos constantes com os periódicos, já exaltados pelo fator de impacto e com experiências de sucesso para internacionalização de seus artigos. As agências de fomento de países como Finlândia, Portugal, Alemanha, Canadá, Reino Unido, Estados Unidos, Polônia, Índia, Bélgica, China, Coreia, França, Holanda, Japão, Noruega e Áustria buscaram o reconhecimento de suas pesquisas não apenas em índices bibliométricos, mas pela importância da pesquisa dentro de suas sociedades. Nesse sentido, a enunciação da ciência aberta trouxe a perspectiva de se olhar a produção científica para as necessidades sociais: o que foi investido na academia precisava ser revertido em benefícios concretos para o bem-estar das pessoas no seu entorno, que deveriam receber todas as vantagens da construção de novos conhecimentos^(25)^.

Esse embate discursivo de dominação do fomento de ciência para o periódico científico *versus* necessidade social, denominada na presente pesquisa de mudança na centralidade de avaliação científica, apresentou-se mais como formação discursiva do que um discurso perfeitamente identificável na terceira década, um fenômeno arqueológico ainda muito desafiador para a análise teórica, devido à contemporaneidade e à necessidade de se observar elementos não discursivos.

Deve-se aceitar que a pesquisa científica e a produção editorial são formas de organização social e que, para manterem a sua entropia, evolução e conquistas de expansão, são necessárias regras e normas. Portanto, a vigilância dentro e fora da APE tornou-se uma necessidade. Desde a modernidade, vigiar e controlar as atividades sociais deixaram de ser uma exclusividade das instituições religiosas ou do Estado, pois as instituições privadas também entraram nesse processo de enunciação discursiva^(29)^. Note-se que os índices bibliométricos de grande importância para os periódicos de instituições públicas, tais como o fator de impacto e o índice H, foram primeiramente delineados por bases de dados de financiamento privado que vendiam esse tipo de bibliometria.

Mergulhadas nessa linha teórica, as sociedades científica e editorial podem ser observadas por seus elementos não discursivos, ou seja, por pessoas, grupos, instituições e empresas que observam seus membros quanto à manutenção do controle do fazer científico, processo social denominado de panoptismo.

A estrutura panóptica volta-se para a regulação científica e editorial, e os membros da APE tornaram-se monitorados por elementos sociais externos durante todo seu período existencial e, em momentos mais recentes, devido às suas conquistas internacionais, tornando-se centro dessa estrutura para outros setores sociais subordinados à sua influência. Quando um indivíduo, grupo ou instituição chega à centralidade dessa estrutura, ele se habilita a controlar as ações de seus subordinados, observando excessos que pudessem perturbar uma ordem esperada^(29)^.

No terceiro decênio da APE, além dos setores sociais acadêmicos e não acadêmicos identificados até então para controle social da ciência, percebeu-se a inclusão do protagonismo de autores e leitores - das enunciações de financiamento, ciência aberta, mídias sociais, ainda incipientes - que provavelmente se prolonga no tempo atual. Assim, o controle social, construído até então por uma estruturação vertical e hierárquica da ordem social (editoração *versus* leitor/autor), também trouxe a ordenação horizontal de contornos econômicos (infraestrutura urbana, instituição, família)^(30)^.

Se antes o diálogo discursivo foi construído entre elementos majoritariamente editoriais, preocupados com as indexações e classificações dos artigos de acordo com o índice H e o fator de impacto, oriundos dos círculos tradicionais (das bases indexadoras comerciais, porém científicas), novos setores sociais não editoriais, como o Google Acadêmico^®^, começaram a disputar a centralidade na estrutura panóptica da comunicação científica. Assim, os índices bibliométricos começaram a ser múltiplos, dependendo de sua fonte, editorial ou não, científica ou não, cuja lógica incluiu *downloads*, compartilhamentos em redes sociais e *likes*, com todos esses índices de desempenho consolidados em mídias sociais e serviços de *streaming* ^(31,32)^.

A enunciação da ciência aberta começou a empoderar autores e leitores e a vigiar e punir também as pesquisas científicas, rompendo a bolha exclusiva dos pareceristas científicos. O panoptismo é um tipo de poder exercido sob a forma de vigilância individual e contínua que permite o controle, o castigo e a recompensa sob a forma de correções. Nesse contexto, as pessoas transformaram-se conforme se adequaram às regras estabelecidas e, consequentemente, modificaram a estrutura panóptica^(33)^.

Se a APE precisou disciplinar diretamente a editoração por meio dos pareceres científicos e dos editores, em consonância com as demandas de instituições externas tradicionais, iniciou-se o confronto/parceria de enunciações das mídias sociais, que incluíram, nessa ordem disciplinar, qualquer indivíduo interessado em ler pesquisas, mesmo não integrante do grupo tradicional de elementos editoriais^(34,35)^. As mídias sociais apresentam enunciações tão importantes na sociedade que elas têm o poder de aumentar a citação bibliográfica, ou seja, as redes sociais influenciam positivamente os índices bibliométricos, efeito tão desejado pelas equipes editoriais^(31)^.

No entanto, essas redes sociais, que são fundamentalmente comerciais, não se resumem apenas a ferramentas de divulgação científica; elas trazem em seu bojo a luta pela centralidade da qualidade editorial e científica, uma crise panóptica em curso, com impacto na construção de uma sociedade disciplinar ligada aos procedimentos de normalização, dos quais as questões são ao mesmo tempo sociais e econômicas^(33)^. Como reação a esse movimento de deslocamento da centralidade panóptica, uma revista médica especializada propôs a criação do cargo de editor de mídias sociais, que teria a função de observar e acompanhar a dinâmica de disponibilização de artigos em plataformas populares e comerciais, fato que demonstrou a aproximação incipiente dos discursos midiáticos aos editoriais dos periódicos, um indício de deslocamento da centralidade panóptica^(36)^.

### Limitações do estudo

Os dados coletados podem ser discutidos com outros editoriais publicados de periódicos nacionais vigentes de enfermagem no mesmo recorte temporal para triangular os depoimentos, reforçando, refutando, ou adicionando discursos novos ao contexto apresentado. Além disso, o aprofundamento na análise dos elementos não discursivos, tais como pessoas e instituições dos primeiro e segundo decênios, poderia fortalecer o estudo da formação discursiva mais completa, porém complexa de ser apresentada em poucas páginas de artigo, merecendo uma dissertação ou tese de doutorado.

### Contribuições para as áreas da enfermagem, saúde ou políticas públicas

A presente pesquisa trouxe mais elementos discursivos e qualitativos para se entender a dinâmica da criação, fortalecimento e manutenção de um periódico de enfermagem, cujos resultados foram além das questões técnicas, editoriais, científicas e bibliométricas, abrangendo visões sociológicas do fazer de uma equipe editorial e sua resposta às demandas sociais, com visões mais externalistas da comunicação científica.

## CONSIDERAÇÕES FINAIS

Os discursos editoriais da APE 1988 a 2017 se basearam na qualidade editorial segundo as demandas sociais de conhecimentos científicos. Compreendeu-se que uma sociedade dinâmica e mutável cobraria alterações na sua trajetória, com adição de novos discursos cada vez mais exigentes, o que demandou dos editores, pareceristas, professores da escola, da pós-graduação e da comunidade científica novas formas de se olhar para a comunicação científica.

Desde a necessidade de enunciar o escoamento da produção científica interna da graduação e da pós-graduação da EPE, a exigência da publicação internacional com pesquisas relevantes e, recentemente, o impacto das mídias sociais em divulgar seus conteúdos, percebeu-se um ambiente composto de diversos elementos discursivos confluentes, antagônicos e complementares.

O controle social da pesquisa e da comunicação científica é um tema de difícil discussão, pois essa análise trabalha com o poder que permeia as relações sociológicas entre os membros de um mesmo grupo, expondo as diferenças nos ganhos sociais embutidos no fazer científico, que se modificam com o tempo e que, para mantê-las, são necessários embates que são silenciados no grupo por meio dos discursos que devem ser defendidos e exaltados.

Diante desse cenário, a APE precisou se adequar para formar o seu discurso oficial, o que possibilitou, ao longo dos anos, mudar sua posição periférica inicial para a central dentro da comunicação científica, corroborando o seu papel panóptico. O panoptismo desenvolvido pela equipe editorial da APE demonstrou a sua luta para se aproximar do centro de vigilância da produção científica. Apesar de ainda estar na periferia das decisões internacionais, onde cumpre o seu papel de receber e de se adaptar aos diversos discursos dos países norte-americanos e europeus, no contexto nacional, ela pertence a um grupo de elite de periódicos de enfermagem e de saúde que se coloca como referencial para as mudanças editoriais necessárias, como se fosse um dos porta-vozes dos periódicos internacionais, trazendo um poder de centralização que demanda muita energia para a manutenção de sua entropia, representada pelos enunciados que circulam sob a sua vigilância. Do ponto de vista foucaultiano, é uma positividade que merece ser destacada em estudos sobre os periódicos de enfermagem no Brasil.
